# Using Markov Chains and Multi-Objective Optimization for Energy-Efficient Context Recognition †

**DOI:** 10.3390/s18010080

**Published:** 2017-12-29

**Authors:** Vito Janko, Mitja Luštrek

**Affiliations:** 1 Department of Intelligent Systems, Jožef Stefan Institute, Ljubljana 1000, Slovenia; mitja.lustrek@ijs.si; 2 Jožef Stefan International Postgraduate School, Ljubljana 1000, Slovenia

**Keywords:** context recognition, optimization, modeling, energy efficiency, Markov chains

## Abstract

The recognition of the user’s context with wearable sensing systems is a common problem in ubiquitous computing. However, the typically small battery of such systems often makes continuous recognition impractical. The strain on the battery can be reduced if the sensor setting is adapted to each context. We propose a method that efficiently finds near-optimal sensor settings for each context. It uses Markov chains to simulate the behavior of the system in different configurations and the multi-objective genetic algorithm to find a set of good non-dominated configurations. The method was evaluated on three real-life datasets and found good trade-offs between the system’s energy expenditure and the system’s accuracy. One of the solutions, for example, consumed five-times less energy than the default one, while sacrificing only two percentage points of accuracy.

## 1. Introduction

Widespread accessibility of wearable sensing devices opens many possibilities for tracking the users who wear them. Possible applications range from measuring their exercise patterns and checking on their health, to giving them location-specific recommendations. For example, we could recognize if the user is walking, running, resting or performing similar activities using accelerometer data. This task was made easier and more practical with the increased use of smartphones, which have many sensors built in. Sensing with multiple sensors, possibly at once, opens additional options for context recognition: recognizing one’s location or contexts such as shopping, traveling or work.

A major limitation of such continuous sensing and context recognition is its heavy toll on the sensing device’s battery life. This is especially relevant for smartphones, which have a very limited battery that must be shared between many applications, but the same limitation applies to basically any wearable device. There is an inherent trade-off between a system’s energy consumption and its recognition quality. Increasing energy savings decreases the recognition quality and vice versa. This issue is often neglected when discussing the design of context-recognition systems; however, it is an essential component if such systems are to be used in practice.

Energy consumption can be reduced by selecting appropriate sensor settings for data collection and/or subsequent data processing. Further optimization can be made by optimizing these settings for every context, as different contexts may require different sensor data or different sensor sampling frequency. Given a list of contexts and possible sensor settings, we aim to find a good assignment of settings to contexts. An assignment example would be: high sampling frequency, when the user is running or walking, but a low sampling frequency when the user is resting. We show that such an assignment should not be done for each context in isolation or the behavior of the system might become unpredictable. To illustrate this, an accelerometer is very good at recognizing walking and resting, while a GPS is very good at recognizing driving. However, if we have only the accelerometer active while walking, driving might never be recognized, and the sensor switch might never occur. Additionally, a misclassification might change sensor settings to ones inappropriate for the current context and cause subsequent classification errors.

To truly take into account all such possible interactions, we would have to run an experiment where we classify all instances in our dataset, using a specific setting for each context and switch between them in runtime. Since there are many (context, setting) combinations that can be tried, this process might be prohibitively time consuming, possibly taking months on large datasets with many possible settings.

We propose a Markov chain model to simulate runtime setting switching and predict what would happen if we used a particular setting for a particular context. Using this model is much quicker than the previously-described experiments, and many setting-to-context assignments can therefore be tried. In addition, we use genetic multi-objective optimization to further increase the efficiency of search for good assignments. Our method provides an accurate estimate of the system behavior under different configurations, requires no hand-picked parameters and works with an almost arbitrary function for evaluating either the system quality or its energy consumption. Furthermore, instead of presenting only one solution, it aims to generate the whole Pareto set of different energy/quality trade-offs, giving the system designer the possibility to make an educated choice between them.

The rest of the paper is organized as follows: In Section 2, we describe the related work; in Section 3 we explain both the Markov chain model and the multi-objective optimization; in Section 4, we describe three real-world datasets used and the energy measurements we made; we then test our approach and report the results in Section 5; and finally, we conclude in Section 6. At the end, there are two appendices, one of them proving theoretical bounds on the problem difficulty (Appendix A) and another that shows how to modify our approach, if we are designing slightly different sensing systems (Appendix B).

This paper builds upon and substantially improves our previous paper published at Ubicomp conference’s UbiMI (4th International Workshop on Ubiquitous Mobile Instrumentation) workshop [1], by providing validation on two extra datasets, adding a more thorough explanation of the method and its testing and including the two previously-mentioned appendices.

## 2. Related Work

To reduce the energy requirements of the system, sensor settings (e.g., the sampling rate or sensor’s duty cycle) can be optimized for a particular recognition task [2,3]. Some authors propose general methods for sensor setting selection, examples being the Shannon sampling theorem [4], which gives a lower bound for the required sampling frequency, determining the sampling frequency by statistically analyzing the properties of the data [5] or heuristically selecting the best sensor subset [6]. This, however, might be suboptimal. We might for example want to have the GPS active at a high sampling rate while the users are driving, but at a very low rate when they are working in the office. This calls for dynamic changes in the settings for both the sensors and for the subsequent processing.

Many adaptive approaches already exist. Lu et al. [7] present a complex pipeline for collecting acceleration, sound and GPS data, suitable for activity recognition. Liang et al. [8] propose hierarchical classification that uses frequency-domain features only when time-domain features prove insufficient. Another approach [9] uses a hand-made flowchart of possible states and lists sensors necessary for detecting transitions between them. Some other works specialize in determining the user’s current location [10,11]. A common problem with these and similar knowledge-based solutions is that they are designed for a specific problem setting. If we were to recognize different contexts using different sensors, we would have to adapt these methods, requiring either a lot of experimentation or expert knowledge. It would be useful to have a method that can be provided with a sensor-rich dataset and can tell which sensors or which sensor setting to use in each context.

A relatively simple solution to the above problem was proposed by Yan et al. [12]. They selected the sensor settings and subsequent processing method based on the last classified activity. For each activity, they found the setting that best recognizes it by testing all the settings on their dataset and then used it when that activity was detected. However, since the selection was made for each activity in isolation, the effect on the whole system could be unpredictable as described in Section 1. Another solution [13] was also based on the last recognized context; based on the current context, it determined the most likely future contexts and selected the sensors that are good at detecting the transitions between them. It relied on parameters that signal the user’s preferences on how much energy to conserve and how much accuracy can be sacrificed. As in the previously mentioned solution, decisions for each context are made in isolation, and the prediction of the accuracy for the whole system is not given. Neither of the proposed solutions model the behavior of the system when the wrong context is recognized.

## 3. Problem Description and Methods

Suppose we have a sensing system that works as follows: the user is in one of the predefined contexts c∈C (e.g., the user’s activity), and the goal of the system is to recognize it. Each context is associated with some setting s∈S. Settings can be which sensors are in use, or what sampling frequency or duty-cycle policy is active, or which feature set is used for classification, and so on. A setting is used as long as the system believes that the user is in a context that corresponds to that setting. When a context change is detected by the system, the setting is changed accordingly. For example: if we are sitting, we might use a low sensor sampling frequency, but when walking is detected, a higher sensor sampling frequency can be turned on.

Given possible settings and contexts, the main issue of this paper is to efficiently find an assignment of settings to contexts (a:C↦S) that generates good trade-offs between the system energy consumption and system quality (for example: the accuracy of the sensing system). What trade-off is considered best is of course up to the system’s designer, but we should strive to present him/her Pareto-optimal solutions from which to choose.

The most straightforward way to determine the optimal assignment of settings to contexts for such a system is to simply try out every assignment. This approach has two obvious problems that make it infeasible for a large number of settings or contexts. First is the exponential number of assignments: if we have $\left|C\right|$ contexts and $\left|S\right|$ settings, we have ${\left|S\right|}^{\left|C\right|}$ different assignments. Given that a sensing system can have many sensors, each having many ways to be configured, we can expect the set of system settings to frequently be large. Second is the time of evaluation of one assignment: one must create appropriate machine learning models and then run through all the data in the dataset, classifying instances while simulating different settings and switching between them accordingly; a process that can be prohibitively slow if we plan on evaluating a large number of assignments.

A common solution is to use expert knowledge to determine sensible assignments and test only those. However, such knowledge is often either not available or not good enough to isolate all good potential assignments, which calls for an alternative, automatic method for solving the task. In Section 3.1, we show how to greatly reduce the evaluation time of a single assignment and then in Section 3.2 how to select a subset of assignments to evaluate.

### 3.1. Reducing the Time of Evaluation

To evaluate as many assignments of settings to contexts as possible, we strive to reduce the time of each evaluation by mathematically estimating the energy consumption of the system and its classification quality and avoid actual experiments. One experiment is considered to be classifying all instances in the dataset using appropriate settings and creating new classification models if so needed for the task. The following two sections describe a naive and a more accurate evaluation model.

#### 3.1.1. Naive Evaluation Model

We start with a simple approach, similar to related work [12,13] in the sense that it models every context in isolation. To use it, we first determine the accuracy of the system using each setting, by experimental evaluation. In these experiments, the same setting is kept throughout, without switching depending on the context. This requires $\left|S\right|$ experiments, exponentially fewer than ${\left|S\right|}^{\left|C\right|}$. Then, to evaluate a specific assignment *a*, the accuracies of each context *c* using the corresponding setting a(c) are summed up, weighted by the proportion of that context in the dataset.

$$\mathrm{Accuracy}\left(a\right)=\sum_{c\in C}p(c)\mathrm{Acc}(c|a(c))$$

a(c): the setting assigned to context *c*.
p(c): the probability of context *c*, estimated by its proportion in the dataset.
Acc(c|s): the accuracy for recognizing context *c*, given the setting *s* is active.

Let us take the example of having two contexts $c_{1}$, $c_{2}$ and two corresponding settings $s_{1}$, $s_{2}$. Additionally, we suppose that using $s_{1}$, we can predict $c_{1}$ with an accuracy of 60% and using $s_{2}$, we predict $c_{2}$ with an accuracy of 100% and that both contexts appear an equal amount of time. In this case, we can make a prediction that this assignment will produce an 80% accurate result.

We can make a similar prediction about the energy consumption, given the information about the energy requirement of each setting.

This evaluation, while easy to compute, models only how the system behaves when the correct setting is active. However, it does not model what happens when a classifier makes a classification mistake and the system subsequently switches to a setting inappropriate for the current context. Additionally, it does not directly take into account what context is likely to appear next and how good is the current setting at classifying it. The naive evaluation is presented both because it is intuitive and as a benchmark to show how modeling the system as a whole reduces its shortcomings.

#### 3.1.2. Markov Chain Evaluation Model

To improve upon the naive model, we propose a Markov chain model for making the performance trade-off estimation. A Markov chain is a stochastic process that moves through predefined states, with the probability of every transition being dependent only on the start and end state. To use this model, we have to make two assumptions about our problem setting. First, that the probability of a transition between contexts depends only on the current context, and second, that the probability of classifying an instance to a context depends only on the current context and the current setting. Both assumptions should hold, at least to a large degree, in most cases of use.

**Figure 1:**
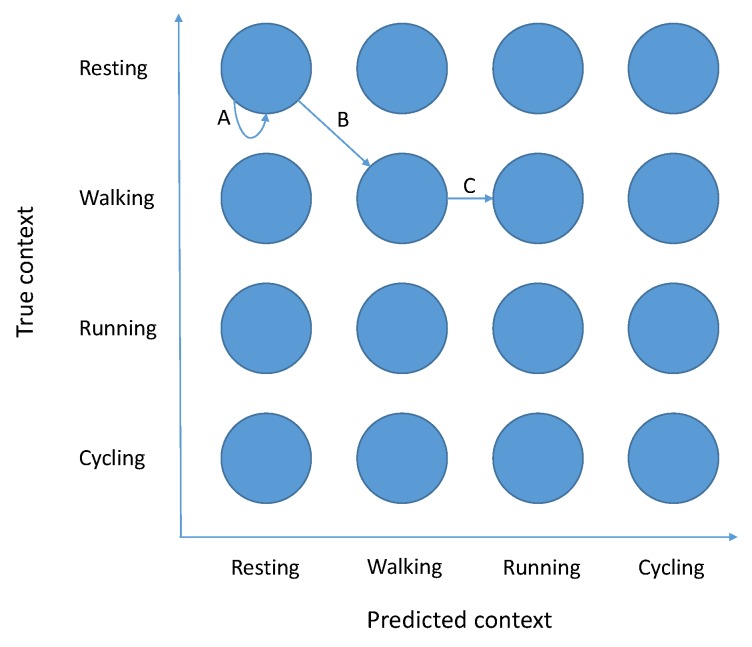
Markov chain, where each state represents a (true, predicted) context of the user. The states are fully connected, including connections to themselves, although most transitions were omitted for clarity. Transition A, for example, models the situation where the user is continually resting, and his/her context is recognized as such. For Transition B, the user started walking, which was correctly recognized. Finally, Transition C implies an incorrect classification of walking as running. The example comes from the E-Gibalec dataset, which is described in Section 4.1.

The key insight is to correctly define Markov chain states, so they (1) contain enough information to calculate transitional probabilities between them and (2) allow us to infer assignment properties from them. We create a Markov chain that has ${\left|C\right|}^{2}$ states. Each state represents a pair (current context, the context the system believes we are in), marked $\langle c,c^{\prime }\rangle$ for short. An example of such a Markov chain can be found in Figure 1.

Our goal is to calculate the “steady-state” of this Markov chain, which gives us the proportion of the time we would spend in each of those states, given infinite (or at least large enough) time. While the structure of the Markov chain model is the same for each assignment, they have different transitional probabilities and subsequently different steady-states. Using this steady-state, we can then make various predictions about the system performance for that assignment that are much better than the ones using the naive approach described in Section 3.1.1.

Similar to the naive evaluation model, we begin by making experiments where for each setting, a confusion matrix is calculated. This is again done without sensor switching and requires $\left|S\right|$ experiments. Additionally, we need the transition probability $T(c_{i},c_{j})$ from each context to each other, which can easily be inferred from the dataset. Finally, we must make the energy consumption estimation per time unit for each setting e(s).

To evaluate a particular assignment, we have to calculate the transition probability from one Markov state to another. They can be calculated from the transition probabilities of the contexts and data from the previously computed confusion matrices. Intuitively: we get in a state $\langle c,c^{\prime }\rangle$ if the context really changes to *c* and if the system classifies this instance into $c^{\prime }$.

(1) $$P\left(\langle c_{1},c_{2}\rangle \to \langle c_{3},c_{4}\rangle \right)=T\left(c_{1},c_{3}\right)C_{a(c_{2})}\left(c_{4}|c_{3}\right)$$

$P(\langle c_{1},c_{2}\rangle \to \langle c_{3},c_{4}\rangle )$: the probability of a transition from state $\left(c_{1},c_{2}\right)$ to state $(c_{3},c_{4}).$
$T(c_{1},c_{3})$: the probability in the dataset that the next context will be $c_{1}$ given that the current one is $c_{3}$.
a(c): the setting assigned to context *c*.
$C_{s}\left(c_{4}|c_{3}\right)$: the probability that the classifier that works with setting *s* will classify an instance to $c_{4}$, if the true context is $c_{3}$.

Having the transition probabilities, we can use the basic Markov chain calculus [14] to calculate the steady-state of the Markov chain. This gives us the amount of time the system will be in each of the Markov states. Since we know how much time we spend in every Markov state, which setting is used in each state and how much energy this setting consumes per time unit, the energy consumption of the whole system can be estimated. Additionally, since the previously calculated confusion matrices give us the accuracy for each setting, we can calculate the accuracy of the whole system in the same manner.

$$\mathrm{Energy}\left(a\right)=\sum_{\langle c,c^{\prime }\rangle \in M}t\left(\langle c,c^{\prime }\rangle \right)e\left(a\left(c^{\prime }\right)\right)$$

$$\mathrm{Accuracy}\left(a\right)=\sum_{\langle c,c^{\prime }\rangle \in M}t\left(\langle c,c^{\prime }\rangle \right)\mathrm{Acc}\left(c|a\left(c^{\prime }\right)\right)$$

*M*: the set of all states in the Markov chain.
a(c): the setting assigned to context *c*.
e(s): the energy requirement of the setting *s* per time unit.
Acc(c|s): the accuracy of the classifier that works with setting *s*, if the true context is *c*.
$t(\langle c,c^{\prime }\rangle )$: the predicted proportion of time spent in state $\langle c,c^{\prime }\rangle$.

It should be noted that many other metrics can be determined from such a model. Example: precision, recall, F-score or the latency of activity-change detection. They can be used instead of the presented accuracy or energy estimations when evaluating the performance of the system using a chosen assignment. Most of those metrics can be calculated directly from the confusion matrix for the whole system, which in turn is trivially calculable from the steady-state.

$${\mathrm{Conf}}_{i,j}=t\left(\langle i,j\rangle \right)$$

${\mathrm{Conf}}_{i,j}$-: the value of the confusion matrix, for correct context *i* and predicted context *j*. Note that all the values are normalized to sum to one; this can easily be remanded by multiplying them by the number of instances, if so needed.

### 3.2. Multi-Objective Optimization

In Section 3.1.2, we showed how to estimate the performance of any individual assignment, and in Section 5, we demonstrated that the estimation is both quick and accurate. As long as the number of assignments is reasonable, we can estimate all of them and then present the Pareto optimal set. This approach scales badly with the increasing number of settings and contexts, as the formula ${\left|S\right|}^{\left|C\right|}$ implies. For large $\left|S\right|$ or $\left|C\right|$, a more efficient search is required. Additionally in Appendix A, we prove that in general, Pareto-optimal assignments cannot be found in polynomial time. However, since we are essentially solving a problem of multi-objective optimization, we can use the methodology from that research field.

We used the NSGA-II [15], a genetic multi-objective optimization algorithm, for the task. We assume, however, that some other similar algorithm could be used in its place. Assignments of settings to contexts are used as the inputs to be optimized. If the settings themselves have a sensible structure (and if we have a huge amount of settings, they are almost bound to have it), we can use this structure to encode the inputs, to make algorithm operations (crossover, mutations) more natural. Example: if our setting is a combination of sensors that can be turned on or off, it can be written as a binary string where one represents an active sensor. We can then concatenate all those strings for each setting that is associated with one of the contexts to get the final input for the algorithm.

The evaluation of any input into energy/quality estimates can be done on-the-fly, when a specific assignment has to be evaluated. This would be excessively time consuming if we used actual experiments instead of evaluations with the Markov chain model.

## 4. Experimental Evaluation

To evaluate our method, we will use three example datasets. The first one is simple and will be used to demonstrate the basic workings of the Markov chain model, while the second and third are more complex and use the Markov chain model and multi-objective optimization in conjunction. At the end of the section, we will also take a look at the energy requirements of the sensors that were used to capture these datasets.

### 4.1. E-Gibalec Dataset

This dataset [16] was recorded on 10 children carrying a smartphone, which was recording acceleration data at a frequency of 50-Hz. The goal was to create a classifier that could recognize four core activities: walking, running, cycling and resting. To test our energy-optimizing method, we chose two sensor settings: the original 50-Hz setting and a down-sampled 5-Hz setting. A hypothesis made with expert knowledge is that having sensors active at high frequency when moving (walking, running, cycling) and low frequency when resting would prove a good trade-off between energy consumption and accuracy.

### 4.2. Commodity12 Dataset

This dataset [17] was recorded on 10 adults who were carrying a smartphone and wearing a chest heart-rate monitor. Each participant captured two weeks of data continuously and hand labeled the following contexts: sleep, work, home, eating, transport, exercise, out (out of house, but not in any of the previous contexts).

The data came from ten different physical and virtual sensors: accelerometer, barometer, light sensor, GPS location, a list of visible WiFi networks, location description by the Foursquare web service [18], sound, time, heart rate and respiration rate. The first eight were measured with the smartphone, while the last two with the heart-rate monitor that was connected to the smartphone via Bluetooth. Features were calculated for each minute of data, and one minute became one learning instance.

While the classification accuracy of the machine-learning models using all the sensors was reasonably high, sensors drained the phone’s battery in less than a day, even if the phone was not used for anything else. This called for energy optimization, which we performed using our approach.

We started by choosing the sensor settings. Every sensor can be either active or not. We also included the option of the sensor in a “duty-cycling” mode, meaning that it is active some set amount of time (one minute in our case) and then inactive for some time (14 min in our case). This list of options may seem short, and surely other options can be added, such as different sensor frequencies, different duty-cycle lengths, etc., but 10 sensors with three options gives us $3^{10}$ settings for any given context. Since we have seven different contexts and a setting must be chosen for each one, this corresponds to ${\left(3^{10}\right)}^{7}$ different system configurations. Since this evaluates to around $10^{33}$ configurations, it is easy to see that any brute-force approach is bound to fail, and any handpicked configuration is likely to be sub-optimal.

The mentioned paper [17] uses a variety of different approaches on how to train and test the context-recognition models, from which we chose the person-dependent approach (a model is trained on the data from a person’s first week and then tested on the data from the second week of the same person). Our method could also be used with any of the other approaches.

### 4.3. Opportunity Dataset

Lastly, we used the publicly available Opportunity dataset [19]. It contains a large number of sensors, tasked to recognize different aspects of the user’s indoor activity. As a subproblem, we chose to recognize which object the user is currently holding in his/her right hand. There were 24 classes, each representing an object held, except for the “none” class, which represented no object in the hand. It is important to note that the class distribution was highly unbalanced and that the “none” class had a representation of 57%. For the recognition task, we used sensors on the user’s body, as well as those placed on some of the objects themselves. Thirty sensor locations were chosen, with an accelerometer, gyroscope and magnetometer at each location. To make a prediction, we started from the baseline provided by the dataset authors [20]. They used nearest-neighbor classification with simple features: mean and standard deviation of the signal. The possible settings in this case were which subset of sensor locations to use. If a location is chosen, all the sensors at that location are used.

While a more detailed energy consumption estimate is made for the first two datasets (Section 4.4), we assumed here for the sake of simplicity that all sensors present have a roughly similar energy consumption. To model their combined consumption, we tested two different approaches. (1) We summed up the individual energy consumptions, with the sum, given the previous assumption, being proportional to the number of active sensors. The final energy requirement of the system is therefore proportional to the average number of sensors used. (2) Since sensors at different locations probably each have their own battery, instead of summing up the sensors, we can determine which location consumes the most energy (by being active the longest) which would make it the bottleneck of our system. By trying to minimize the longest active time, we would expect to get configurations that switch between the sensors as often as possible, to reduce the load on each individual one.

### 4.4. Energy Consumption

To model the energy consumption of a system, one must first understand how it is affected by different sensor settings. This interaction is in many cases given by the sensor manufacturer. In other cases, one could make some rough estimate, for example count the average number of active sensors to achieve a good enough energy estimate. In the case of the E-Gibalec and Commodity12 datasets, we deal with a smartphone, the energy estimation of which is more complicated. This is partially because not all the phone’s sensor details are available and partially because different sensors’ energy requirements do not add up in a linear way. An example of the latter is: the sum of the energy required by the phone with the accelerometer active and the phone with the gyroscope active is much larger than the energy required by the phone that has both the gyroscope and the accelerometer active at the same time.

We did some simple measurements for the purpose of presenting our method, but the interested reader may consult other papers on how a smartphone’s energy consumption works in more detail [21,22]. All our measurements were done on a Samsung Galaxy S4 device (without a SIM card), using a multimeter attached directly to the device’s battery contacts. We measured the current, which is proportional to the energy consumption, given a relatively constant voltage.

First, we made a baseline measurement, with an “empty” process that only kept the processor from sleeping. Second, we tested how increasing the accelerometer frequency affects the battery consumption. Last, we estimated the energy requirements for different combinations of sensors being active. Since the individual-sensor energy consumptions do not add up linearly, we had to try every combination. Fortunately, this process was simplified by the following factors: (1) for both the heart rate and respiration rate, only the Bluetooth connection is required, and its power consumption is the same with one or two data streams; (2) having one, two or all of the following sensors—accelerometer, light and pressure—resulted in a very similar energy consumption, perhaps because they share many resources to operate; (3) Foursquare is “free” if WiFi and GPS sensors are active, and it costs as those two are otherwise (it needs both to work); (4) the time “sensor” is free; (5) GPS added an extra 40 mA on average regardless of other sensors. This narrowed the number of required combinations to test down to 16. The results are listed in Table 1.

To estimate duty-cycle energy consumption, we used a simple approach of taking the proportion of the time the sensors are active, multiplied by the sensors’ energy consumption and then adding the proportion of time they are sleeping multiplied by the sensor combination that excludes “duty-cycled” sensors. In all the estimations, we assumed that turning a sensor on or off requires no additional power. This is not always the case, GPS being a notable exception, but serves to simplify our energy consumption model.

## 5. Results

### 5.1. E-Gibalec

**Figure 2:**
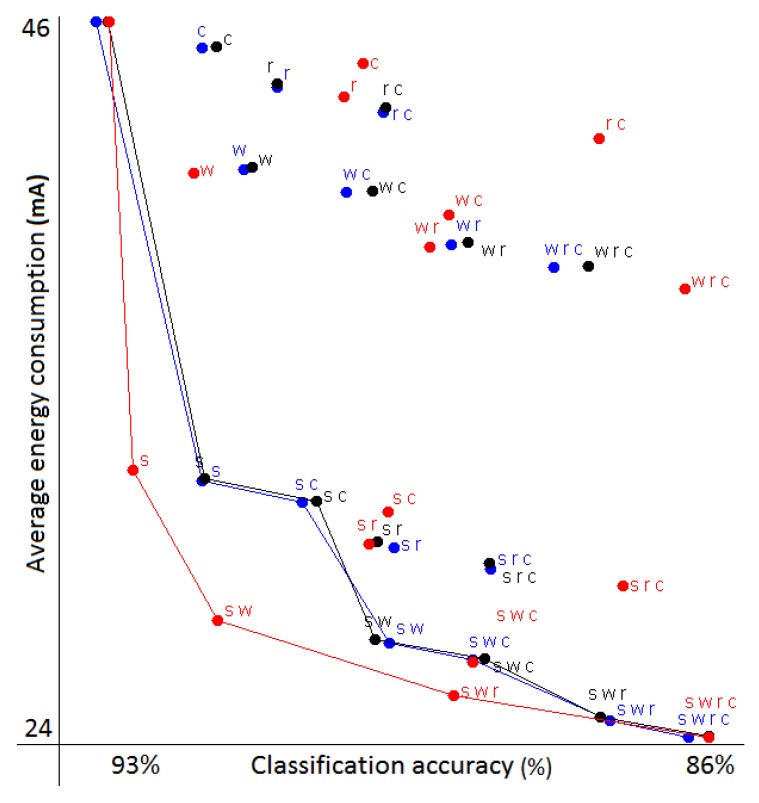
The black points are the real trade-offs; the blue points are simulated with Markov chains; and the red points are simulated with the naive model. The Pareto fronts are drawn using the corresponding colors. The points are labeled with letters that correspond to the activities where the low frequency was used: w, walking; r, running; c, cycling; s, rest. Note that the Pareto front is usually drawn with step-shaped lines, but we connected points directly for greater clarity. The red Pareto front might look the best, but this is a consequence of overly optimistic estimation, not of better solutions.

This example features only 16 different possible assignments, since we have four contexts and two sensor settings. To evaluate if we can accurately predict the performance of all of them, we first estimated the trade-offs using both the Markov chain model and the naive model. We then ran the actual experiments we were estimating, switching classifiers and sampling frequencies during the runtime. All three sets of results are plotted in Figure 2. In addition, we calculated the mean absolute error (MEA) between the predicted accuracy/energy and the real one determined by the experiments. The results are shown in Table 2.

The trade-offs in Figure 2 are marked with letters that correspond to the activities where the low frequency was used. The case where only rest was used with the low frequency (marked as ‘s’) can be considered as one of the best trade-offs between the energy gained compared to the accuracy lost, as we already predicted when describing the data. We also plotted the Pareto front that connects the non-dominated solutions (a solution is dominated if another solution exists that is better in both the classification quality and energy consumption). We can see that the Markov chain simulation points correspond very closely to the values of real experiments. On the other hand, naive simulation tends to be overly optimistic, which is generally the case, given it models only one kind of possible classification error.

We also explored what happens if the underlying activity distribution in the dataset changes. This can easily be simulated by modifying the transition probabilities of the Markov chains. In this case, it turns out that while the values themselves change drastically, the overall shape of the Pareto front remains similar. This means that the best simulated trade-off likely remains the best with most other activity distributions. Such simulations are handy if the underlying activity distribution is unknown or if we are interested in seeing if the energy requirements exceed the application limits if some condition changes. Note that no additional experiments with the actual data were needed to generate this information, which is an additional benefit of our method.

While all 16 possible assignments could easily be tried experimentally, we used this simple example to show that our Markov chain model makes accurate predictions that can replace the actual experiments and can be evaluated roughly 12,000-times faster. More realistic use cases are presented in the following two sections.

### 5.2. Commodity12

**Figure 3:**
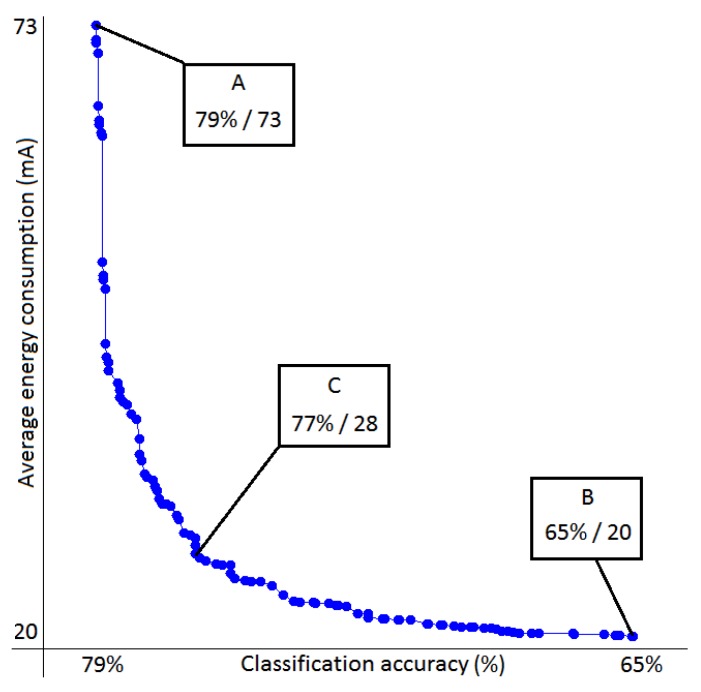
Non-dominated set of assignments evaluated in the Commodity12 experiment. Three points of interest were labeled, and their value is displayed as the classification accuracy (%)/average energy consumption (mA).

In this case, the number of assignments was much too large to experimentally try all, too large even to evaluate all using the Markov chains. We therefore used the NSGA-II algorithm to find a set of non-dominated solutions approximating the Pareto front and used Markov chains for the evaluation of the solutions that the algorithm was iteratively selecting. The resulting set can be found in Figure 3.

#### 5.2.1. Accuracy Evaluation

We start again by estimating the MAE between the predicted values and those we got in real experiments with runtime sensor and data switching. Three hundred random assignments were chosen to be evaluated, and the results are listed in Table 3. The F-score was added to demonstrate that other metrics of classification quality can be predicted and used instead of the accuracy.

In all cases, the Markov chain model outperformed the naive one and was very close to the actual values. Next we evaluated the same error on the final non-dominated set (seen in Figure 3). There was a reasonable concern that predictions on this set would be more error-prone, as overly optimistic prediction about the system performance might place that assignment in the non-dominated set. The results are shown in Table 4 and disprove this hypothesis.

#### 5.2.2. Non-Dominated Set

The solutions presented in Figure 3 can be provided to the sensing system designer, who can decide which trade-off is the most suitable for the system. Point A shows an assignment that performs as well as the original system with all the sensors turned fully on. However, it consumes only half as much as the original (which consumed 140 mA). Point B has only the sensor “time” active. As these data are essentially free, the sensing system consumes as little energy as the baseline case. Even so, it performs at a 65% accuracy, much higher than the majority classifier would (the majority class occurs in 46% of the cases). Since the training and test data came from the same person, it is easy to explain why the time of the day might be an informative feature. Lastly, we have Point C, which looks like a reasonable trade-off. It consumes little energy, but loses negligible accuracy compared to the best case. It accomplishes this by discarding the sound entirely and using the GPS sparsely. All the other sensors are almost always in the “duty-cycle” mode, which can be considered reasonable as the daily contexts in this dataset do not change often. The two most frequent contexts “home” and “work” can also be recognized solely by using the list of visible WiFi networks.

#### 5.2.3. Time Evaluation

We have shown that we can get good assignments using our method; now, we also show the time that was saved using it. The Markov chain model performs roughly 37,000-times faster on average on the Commodity12 dataset than the average experiment, needing 1.5 ms for a single evaluation in comparison to 55,000 ms for an experiment (time of building a model excluded). A much greater number of assignments can therefore be evaluated in a given time, giving us better chances of finding good solutions.

The benefits of multi-objective optimization compared to exhaustive search have been thoroughly demonstrated in the past, and in this domain, it is no different. Solutions shown in Figure 3 were found after evaluating 100,000 assignments, magnitudes less than the $10^{33}$ assignments in the search space.

### 5.3. Opportunity

**Figure 4:**
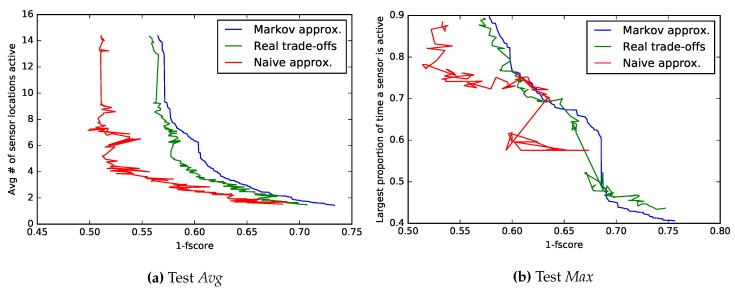
Non-dominated set of assignments evaluated in two Opportunity experiments. Both minimized F-score and an energy metric: on the left, the average number of active sensor locations is minimized; on the right, we minimize the proportion of time the most frequent sensor location is used. The blue line signifies the found non-dominated set using the Markov chain model. The same assignments were then tested with a real experiment (green) and with the naive model (red). Note that blue and red evaluations do not form a non-dominated set. The goal of the approximation is to be as close as possible to experimentally-achieved values (green); as in other datasets, the naive approximation is overly optimistic in almost all cases.

The Opportunity dataset was tested similarly to the Commodity12 dataset, with similar conclusions. We made two tests (subsequently named *Avg* and *Max*), with different optimization functions described in Section 4.3, demonstrating the flexibility of our method. The non-dominated sets found in each case are plotted (Figure 4) in addition to a comparison with naive approximations and experimentally-acquired trade-offs. Differences in accuracy between both approximations are also listed numerically in Table 5 and again show the Markov model as the better performing one. Note that while all F-scores seem low, the problem itself has 24 different classes, which are being classified using simple features only.

#### 5.3.1. Non-Dominated Set: Test *Avg*

Among the configurations found by our method, there was one that worked as well as the original, but used on average 14 locations of sensors instead of 30. Additionally, another configuration sacrificed 0.02 in F-score to reduce the average number of locations used down to seven. Not entirely surprisingly, most solutions rely heavily on the sensors placed on the user’s right arm and back in addition to those sensors that are placed on the objects currently in use. Example: if the system suspects that the spoon is being used, it activates sensors on the spoon to confirm it. In addition, the objects that are often used in conjunction are also checked, e.g., if bread is suspected to be held, the motion of the knife is also checked.

#### 5.3.2. Non-Dominated Set: Test *Max*

When trying to find out how much time the most frequent sensor location was active, it is really important to model the system as a whole, instead of every context individually; as seen by the quite wrong shape of the naive approximation in Figure 4. Reasonable solutions have the most used sensor location working around 65% of time, increasing its battery life by roughly a third. Note that since 57% of the instances belong to the base class “none” and some sensors are always active when “none” is recognized, one would assume that some sensor location is going to be active at least that proportion of the time. This is true for the Naive approximations; Markov chains, however, also model misclassifications, enabling them to find configurations that wrongly classify the “none” class, increasing energy gains even further in exchange for a substantial classification quality loss.

## 6. Conclusions

The results in this paper show that using Markov chains, we can accurately and quickly predict the behavior of a sensing system that is switching between its settings based on the current context. This prediction takes into account the system as a whole and in prediction accuracy outperforms a simpler model that looks at every context in isolation (similar as what was done in other works).

Combining the Markov chain model with an efficient search, implemented using a multi-objective genetic algorithm, provides a powerful tool for optimizing the energy needs of the system while maintaining its accuracy. The method is simple to use, requiring no parameters set in advance and no expert knowledge except for the list of possible settings. It is fast and can work with almost any classification quality or energy consumption metric. It is best used in cases where adapting settings to contexts is sensible, with many different settings to choose from and no clear expert-knowledge solution.

Using this approach, we found good solutions on all three tested datasets. One of the solutions for the Commodity12 dataset was estimated to consume five-times less energy in exchange for 2% accuracy loss, better than any solution we found using expert knowledge alone. We plan on implementing it and deploying it in real life.

The limitations of the system that we will attempt to tackle in the future work include that both contexts and sensor settings must be discrete (instead of, for example, using all duty-cycle lengths). Possible sensor settings must also be provided as an input, and we are looking into ways to automate the process and help the developer decide on the sensor settings to pick. Since the Markov chain model takes misclassifications into account, it should never suggest a configuration where a single misclassifications causes a long chain of future mistakes; by for example, turning off a key sensor for detection of the current context. However, additional safety mechanisms can be implemented to address unforeseen circumstances; for example, turning on all the sensors periodically or if the classifier confidence drops under a certain threshold.

The future work will also include trying to use a similar method, but adapt the system “on-the-fly”, based on the current user behavior. Adopting transitional probabilities between contexts and other similar user-specific parameters would allow for further optimization of energy consumption. Another interesting direction would be trying to configure the system to work on multiple context recognition tasks with different requirements simultaneously (e.g., recognizing the user’s activity, as well as stress level).

## Figures and Tables

**Table 1:** Energy cost of different sensor combinations measured in mA. Sensors that were active were labeled: A, accelerometer (50 Hz), A (5 Hz), accelerometer (5 Hz); S, sound; B, Bluetooth; W, WiFi.

Combination	Current (mA)	Combination	Current (mA)	Combination	Current (mA)
/	20	A (5 Hz)	24	A	46
B	45	W	30	S	55
A, B	52	B, W	75	B, S	68
A, W	70	A, S	61	W, S	84
A, B, W	74	A, B, S	70	B, W, S	90
A, W, S	85	A, B, W, S	100		

**Table 2:** The average prediction error, made on predicting the energy consumption and classification accuracy for the naive and Markov chain model. The values are given in % with respect to the range of possible values for the accuracy and energy consumption (see Figure 2).

	Accuracy	Energy
Markov	2.03	0.35
Naive	12.82	1.83

**Table 3:** The average prediction error of random assignments, made on energy consumption, F-score and accuracy for the naive and Markov chain model.

	Accuracy (%)	F-Score	Energy (mA)
Markov	3.0	0.02	1.4
Naive	5.2	0.04	4.7

**Table 4:** The average prediction error of assignments in the non-dominated set, made on energy consumption, F-score and classification error for the naive and Markov chain model.

	Accuracy (%)	F-Score	Energy (mA)
Markov	2.3	0.03	1.1
Naive	5.8	0.09	4.9

**Table 5:** The average prediction error when predicting the energy requirements and F-score of assignments in the non-dominated set, for both tests, made by the naive and Markov chain model.

	Test *Avg*		Test *Max*	
	F-Score	Energy	F-Score	Energy
Markov	0.02	0.06	0.01	0.02
Naive	0.05	0.09	0.06	0.06

## Appendix A. Theoretical Bounds

In this Appendix, we show that the problem of assigning settings to contexts cannot be solved in polynomial time, not even if we substantially simplify the problem. One could assume that improving the setting (by adding sensors, increasing sensor frequency, etc.) will increase the system quality (ex. accuracy), but decrease its energy efficiency, and that therefore a systematic and efficient way for constructing the Pareto front exists. This, however, is not the case.

First, the assumption does not hold: sometimes, giving a method more data decreases its quality, especially if the new data are not relevant for the classification problem at hand. Second, even if it held, it would not be enough: we will show that by constructing a specific subproblem that is well behaved, and we will show that the subproblem can be translated to the well-known knapsack problem. Thus, we will prove that the specific subproblem is NP hard and, as a consequence, that the general version of the problem is NP hard.

Consider the system *SimplifiedSystem* with the following properties.

1. The sensing system is composed of different sensors that are either on or off (*R* = {$r_{1}$, $r_{2}$, …, $r_{n}$})
2. Each setting *s* is defined as a set of indices of the currently active sensors (example: $s=\left\{r_{1},r_{3},r_{6}\right\}$)
3. Each sensor adds a fixed amount to the system quality and to the system energy consumption, regardless of what other sensors are currently active, depending only on which context c∈C it was added to (adding a sensor is more beneficial for recognizing some contexts than others).

Example: If the accelerometer is activated when walking, the accuracy of the whole system goes up by 5%; if it is added to running, the accuracy goes up by 10%; if it is added to both, the accuracy goes up by their sum: 15%. This third item on the list greatly simplifies the problem and allows us to calculate the quality and energy for a single assignment in the following manner.

(A1) $$Quality=\sum_{c\in C}\sum_{r\in s(c)}q\left(c,r\right)$$

(A2) $$Energy=\sum_{c\in C}\sum_{r\in s(c)}e\left(c,r\right)$$

s(c)-: the setting (set of indices) corresponding to context *c*.
q(c,r),e(c,r): quality and energy gains, given context *c* and sensor *r*.

Since every subset of *R* defines its own setting and since each context must have a setting assigned, it trivially follows that:

**Theorem** **A1.** In the SimplifiedSystem, there are $2^{\left|R\right|}$ settings. Exhaustive search requires checking ${\left(2^{\left|R\right|}\right)}^{\left|C\right|}\;=\;2^{\left|R\right|\left|C\right|}$ different assignments.

We can interpret every (context, sensor) combination as an item with value q(c,r) and weight e(c,r). This way, we get $\left|R\right|\left|C\right|$ different items. Mimicking the knapsack problem, we can put some of the items in a knapsack. Every assignment of settings to contexts corresponds to placing all the corresponding items in the knapsack. This correspondence is bijective: for each knapsack filling, there is an appropriate unique assignment. In the original knapsack problem, we have to find the best value given a weight limit. Let us define *ParetoKnapsack* as a problem where we have to find the best value for each possible weight limit (a Pareto front of value against weight). This is a strictly harder version of the problem and is therefore also NP hard.

The problem of assigning settings to contexts in *SimplifiedSystem* can therefore be directly translated to the *ParetoKnapsack* problem. The worst case for the time complexity of the knapsack problem is O($2^{\mathrm{number}\;\mathrm{of}\;\mathrm{items}=\left|R\right|\left|C\right|}$). It therefore follows:

**Theorem** **A2.** Any method for finding Pareto-optimal assignments for the SimplifiedSystem has the time complexity of O($2^{\left|R\right|\left|C\right|}$) in the worst case. This is no better than the exhaustive search.

We have shown that even the simplified version of the problem cannot be efficiently solved if we desire an optimal solution, which implies that neither can be the general version. This justifies our use of heuristic methods to find near-optimal solutions.

## Appendix B. Different System Schemes

In this work, we considered a scheme where each context is assigned a setting. Those settings then change as different contexts are recognized. However, by adapting a Markov chain model, similar system schemes can be constructed and evaluated. This Appendix provides an example:

Consider a system that uses most of the sensor data to recognize the user’s context, but then having recognized it, it uses limited data to determine if the context remains unchanged. Example: after recognizing that the user is sitting, only a low-sampling accelerometer is required to determine the persistence of the activity. After movement is detected, a general classifier starts working again to recognize the next context. This is in essence similar to the scheme proposed in our work and can be modeled in a similar fashion.

“Transition” context is added to the list of contexts and represents the state after the system detects a context change and starts the general classifier. “Transition” context is marked with ‘t’ in all cases, while all other letters for states imply non-transitional context. A typical sequence of (true, predicted) contexts would be as follows: $\langle c_{1},c_{1}\rangle \to \langle c_{2},t\rangle \to \langle c_{2},c_{2}\rangle$.

Transitional probabilities are slightly more complicated, as now we have two types of contexts, but the general concept and intuition behind them remains the same. The following equations list the transitional probabilities and replace Equation (1) in Section 3.1.2. Equations (A3) and (A4) show the transitional probability of zero, since the transitional context is always followed by a “real” one, and transition from one context to another always goes through the transitional context. This should give the reader an idea of how to approach and model similar schemes.

(A3) $$P(\langle c_{1},t\rangle \to \langle c_{3},t\rangle )=0$$

(A4) $$P(\langle c_{1},c_{2}\rangle \to \langle c_{3},\neg c_{2}\rangle )=0$$

(A5) $$P\left(\langle c_{1},c_{2}\rangle \to \langle c_{3},t\rangle \right)=T\left(c_{1},c_{3}\right)C_{a(c_{2})}\left(\neg c_{2}|c_{3}\right)$$

(A6) $$P\left(\langle c_{3},t\rangle \to \langle c_{4},c_{5}\rangle \right)=T\left(c_{3},c_{4}\right)C_{t}\left(c_{5}|c_{4}\right)$$

(A7) $$P\left(\langle c_{1},c_{2}\rangle \to \langle c_{3},c_{2}\rangle \right)=T\left(c_{1},c_{3}\right)C_{a(c_{2})}\left(c_{2}|c_{3}\right)$$

$\neg c_{2}$: context different than $c_{2}$ or *t*
a(c): setting assigned to context *c*
$T(c_{1},c_{3})$: the probability in the dataset that the next context will be $c_{3}$ given that the current one is $c_{1}$.
$C_{s}\left(c_{4}|c_{3}\right)$: the probability that a classifier that works with setting *s* will classify an instance to $c_{4}$, if the true context is $c_{3}$.
Note: when $c_{i}$ and $c_{j}$ are listed in the same equation, *i* can equal *j*, unless otherwise stated.
